# Novel five-phase model for understanding the nature of misophonia, a conditioned aversive reflex disorder

**DOI:** 10.12688/f1000research.133506.2

**Published:** 2023-08-04

**Authors:** Thomas Dozier, Nathanael Mitchell

**Affiliations:** 1 Misophonia Institute, Livermore, CA, 94550, USA

**Keywords:** anxiety, emotional development, clinical, learning, neuropsychology, psychopathology, psychophysiology

## Abstract

**Background:** Misophonia is a recently identified condition in which a person perceives a subtle stimulus (e.g., eating sounds, hair twirling) and has an intense, negative emotional response. Misophonia cannot be classified with established nosological systems.

**Methods:** We present a novel five-phase model of misophonia from a cognitive-behavioral framework. This model identifies a learned reflex of the autonomic nervous system as the primary etiology and maintenance of misophonia. Phase one is anticipatory anxiety and avoidance. Phase two is a conditioned physical reflex (for example, the tensing of calf muscles) that develops through stimulus-response Pavlovian conditioning. Phase three includes intense negative emotional responses and accompanying physiological distress, thoughts, urges, and emotion-driven behavior. Phase four is the individual’s coping responses to emotional distress, and phase five is the environmental response and resulting internal and external consequences of the coping behaviors. Each phase helps explain the maintenance of the response and the individual’s impairment.

**Results:** Anticipatory anxiety and avoidance of phase one contributes to an increased arousal and awareness of triggers, resulting in increased severity of the trigger experience. Both the Pavlovian-conditioned physical reflex of phase two and the emotion-driven behavior caused by the conditioned emotional response of phase three increase with *in vivo* exposure to triggers. Phase four includes internal and external coping behaviors to the intense emotions and distress, and phase five includes the consequences of those behaviors. Internal consequences include beliefs fiveand new emotions based on environmental responses to anger and panic. For example, the development of emotions such as shame and guilt, and beliefs regarding how ‘intolerable’ the trigger is.

**Conclusions:** We assert misophonia is a multi-sensory condition and includes anticipatory anxiety, conditioned physical reflexes, intense emotional and physical distress, subsequent internal and external responses, and environmental consequences.

## Introduction

Ivan Pavlov was awarded the Nobel Prize in Physiology in 1904 ( Nobel Prize, 2022). For over a century extensive research has been conducted on Pavlovian or classical conditioning. Yet a newly identified condition known as misophonia illustrates a substantial deficiency in our understanding of Pavlovian conditioning; a condition which is fundamentally a Pavlovian conditioned reflex, likely affecting hundreds of millions of people worldwide ( Kılıç *et al.,* 2021; Cash, 2015).

Misophonia was first identified in 1997 by audiologist Marsha Johnson as soft (or selective) sound sensitivity syndrome ( Bernstein *et al.,* 2013; Neal and Cavanna, 2012; Yılmaz and Hocaoǧlu, 2021), and later named by Jastreboff and Jastreboff (2002). For over a decade it was exclusively investigated and treated as a poorly understood hearing condition in which common specific sounds caused a person to experience intense negative emotions and distress ( Kılıç *et al.,* 2021; Swedo *et al.,* 2022). Misophonia was (and sometimes still is) grouped with hyperacusis and referred to as “decreased sound tolerance” ( Jastreboff and Jastreboff, 2002). Treatment/management was primarily provided by audiologists with ear-level sound generators and methods developed for tinnitus ( Jastreboff and Jastreboff, 2014). Awareness of misophonia beyond the audiologist community was greatly enhanced in 2011 with a New York Times article titled, ‘When a Chomp or a Slurp is a Trigger for Outrage’ ( Cohen, 2011). Soon after, understanding of the condition began to grow as research into the condition began.

Misophonia is now commonly known as a condition whereby a person perceives an innocuous auditory or visual stimulus (e.g., hearing chewing, seeing hair twirling) and has an immediate, intense, negative emotional response, including physiological distress, and strong behavioral responses ( Brout *et al.,* 2018; Claiborn *et al.,* 2020; Dozier *et al.*, 2017; Edelstein *et al.,* 2013; Jager *et al.,* 2020; Rosenthal *et al.,* 2021; Schröder *et al.,* 2013; Swedo *et al.,* 2022; Wiese *et al.,* 2021). A misophonia trigger is based on specific pattern, meaning, and experience of the individual with that stimulus, rather than the intensity or frequency components of the stimulus ( Brout *et al.,* 2018; Jastreboff and Jastreboff, 2014; Swedo *et al.,* 2022). Although oral and nasal sounds are the most prevalent triggers, each person has their own unique set of triggers. Emotional responses to triggers commonly include irritation, anger, disgust, anxiety, and escape ( Brout *et al.,* 2018; Swedo *et al.,* 2022). Experiencing a misophonic trigger is often very distracting and causes dysregulation of thoughts and emotions which can impair social, occupational, or academic functioning. Severity of misophonia can vary from mild to debilitating. Onset of misophonia is typically in late childhood or early teens, but studies report onset in adulthood in 9% ( Rouw and Erfanian, 2018) and 10% ( Claiborn *et al.,* 2020) of study participants.

Misophonia is a clinically significant mental health issue, with prevalence estimates in adults based on community samples reported at 12.8% ( Kılıç *et al.,* 2021) and 13.5% ( Cash, 2015). Surveys of clinical populations such as those seeking help from a psychiatric or psychological clinic have been reported at 35% ( Ferrer-Torres and Giménez-Llort, 2021) and 66% ( Quek *et al.,* 2018), respectively. Misophonia is a poorly known condition in part because it was recently identified and is not included in the Diagnostic and Statistical Manual of Mental Disorders, 5 ^th^ Edition (DSM-5; American Psychiatric Association, 2013) or International Classification of Diseases, 11 ^th^ Edition (ICD-11; World Health Organization, 2019/2021).

There is a lack of consensus on both the etiology of misophonia and the putative mechanisms of the behaviors of the misophonic response, in part because of the limited supporting research for any theory of misophonia, with the exception of functional magnetic resonance imaging (fMRI) studies. Several neurological defect mechanisms and behavioral models have been proposed. However, theories generally lack specificity, and tend to be based on limited correlational data, work with patients, and the researcher’s worldview. Møller (2011) postulated that misophonia is caused by an anatomical anomaly in central nervous system regions (e.g., the inferior part of the temporal lobe), and Palumbo *et al.* (2018) suggested a breakdown in interaction processes between the limbic system and classical and non-classical auditory pathways. Furthermore, Edelstein *et al.* (2013) proposed that such a distortion of connections could cause a form of “sound-emotion synesthesia.”

Jastreboff and Jastreboff (2002) presented a theory of the neural systems and mechanisms involved in misophonia. The same model and connections are used in their models for tinnitus, hyperacusis, and misophonia, identifying the primary brain areas and strength of interconnection activation for each condition. This model shows interconnections between the auditory subconscious for detection and processing, the auditory and other cortical areas for perception and evaluation, the limbic system for emotions, and the autonomic nervous system all of which connect to ‘reactions’. The authors proposed that the primary areas of the brain for misophonia are the “auditory and other cortical areas for perception and valuation” and the limbic system (p.77). They theorize that misophonia might involve “abnormally strong reactions of the autonomic and limbic systems resulting from enhanced connections between the auditory, limbic and autonomic systems, or enhanced reactivity of the limbic and autonomic system to sound” ( Jastreboff and Jastreboff, 2002, p.77). Additionally, they noted that “connections involved in misophonia are controlled by conditioned reflexes principles” ( Jastreboff and Jastreboff, 2002, p.77). Activation of the autonomic nervous system (ANS) is an important component of this model, but the ANS is not identified as a primary brain area responsible for misophonia. This implies the misophonic trigger stimulus elicits the emotions (limbic system) and that, in turn, elicits the activation of the ANS. This theory attributes the etiology of misophonia to conditioning, and the authors recommend treatment with tinnitus retraining therapy (TRT) which includes counterconditioning/active extinction (i.e., pairing weak auditory triggers with positive sounds) to reduce the conditioned reflexes of misophonia. Although this theory was presented only for auditory triggers, it could be extended to other sensory modality triggers.

Kumar *et al.* (2017, 2021) proposed neural mechanisms for misophonia. Using fMRI, they identified significantly higher activity in structures involved with regulation and associative learning of emotions (i.e., ventromedial prefrontal cortex [vmPFC], and anterior insula cortex; 2017) among misophonic individuals. Furthermore, there were between-group differences in the emotional response to trigger stimuli, with the misophonic group showing increased vmPFC activation compared to controls. Given the crucial role of the vmPFC in the development of reflexive positive or negative affect responses to initially neutral stimuli, Kumar (2015) asserted the emotional response of misophonia appears to be a learned emotional reflex. Additionally, Kumar (2015) identified enhanced myelination in the misophonic group in the interconnection of these structures and the amygdala and hippocampus, implying enhanced neuron functioning. These findings were confirmed in a subsequent independent fMRI study ( Schröder *et al.,* 2019). Kumar *et al.* (2021) later proposed a motor basis for misophonia after reporting the involvement of mirror neurons for oral/facial movement of individuals experiencing a trigger. Mirror neurons are responsible for common copying behaviors (i.e., monkey-see, monkey-do). One limitation of this theory is that it does not seem to apply to non-human triggers (e.g., rooster crowing, motorcycle sound). A second limitation of both studies is that the research is correlational, so it is unclear whether these neural correlates are a cause or result of misophonia.

Most other theories consider misophonia a phenomenon that develops through associative (i.e., classical conditioning) and/or non-associative (i.e., sensitization) learning ( Bernstein *et al.,* 2013; Cowan *et al.*, 2022; Dozier, 2015b; Jastreboff and Jastreboff, 2002; Palumbo *et al.,* 2018; Schröder *et al.,* 2013; Webber and Storch, 2015; Wu *et al.,* 2014). Various explanations for the conditioning of misophonic responses have been proposed. For example, some have argued that misophonia develops as a physical reflex where the trigger (e.g., eating sound) is the conditioned stimulus (CS), with anger, irritation, or stress as the unconditioned stimulus (US; Palumbo *et al.,* 2018). Similarly, it has been proposed that with some individuals there is an US of negative interpersonal interactions with a specific family member and an unconditioned response (UR) of anger due to rigidity and judgement, so the otherwise neutral sounds of that family member (e.g., chewing) becomes the CS and anger becomes the conditioned response (CR; Cowan *et al.,* 2022).

Operant conditioning is broadly recognized as the process that leads to avoidance of triggers by virtually all individuals with misophonia. Citations for the avoidance component of misophonia might include all the above references.

Many successful treatment cases applied components of a cognitive-behavioral framework (CBT) and acceptance based (acceptance and commitment therapy, ACT) interventions ( Aazh *et al.,* 2019; Alekri and Saif, 2019; Altýnöz *et al.,* 2018; Bernstein *et al.,* 2013; Cecilione *et al.,* 2021; Dozier, 2015a, 2022; Dover and McGuire, 2021; Hocaoǧlu, 2018; Lewin *et al.,* 2021; McGuire *et al.,* 2015; Muller *et al.,* 2018; Reid *et al.,* 2016; Robinson *et al.,* 2018; Roushani and Honarmand, 2021; Schneider and Arch, 2017; Tonarely-Busto *et al.,* 2022; Vanaja and Abigail, 2020). The success of misophonia treatment trials using CBT and ACT components also provides support for the theory that misophonia is a learned condition ( Frank and McKay, 2019; Rabasco and McKay, 2021; Schröder et al., 2017). Recently, Cowan *et al.* (2022) proposed a psychological model of misophonia which includes a recurring, circular interconnection of distress from sound, rigidity regarding sounds, and increased awareness of sounds. This contributes to the development of the misophonic responses and continually strengthens and maintains them. They state that the initial development of misophonia occurs through classical and operant conditioning as follows: 1) predisposing context (e.g., negative affect), 2) sound made, 3) rigidity (i.e., judgment the sound should not be made), 4) initial negative reaction (e.g., anger), 5) increased awareness of the sound, 6) negative association with sound (i.e., classical conditioning), and 7) avoidance which develops from operant conditioning and generalizes to other stimuli. They propose a treatment which they call “experiential acceptance and stimulus engagement” (EASE, Cowan *et al.,* 2022, p.1) based on this model.

Dozier (2015b) proposed a behavioral model of misophonia. He theorized that the etiology of misophonia is a Pavlovian-conditioned initial physical reflex (IPR), which is typically a skeletal muscle response, but could be other responses (e.g., stomach constriction, nausea, intestine constriction, esophagus constriction, sexual arousal, urge to urinate, etc.). In this model, the trigger is the conditioned stimulus (CS) and the muscle response (IPR) is the conditioned response (CR). Dozier proposed that the IPR develops through stimulus-response conditioning, where there is a repeating stimulus (CS) and a temporally occurring muscle response (CR). For example, a person could hear someone sniff and think, ‘I don’t want to catch their cold’, and hold their breath. The sniff is not an unconditioned stimulus (US) and holding one’s breath is not an unconditioned response (UR). But with repeated sniffing and breath holding, the person could develop a reflex response (CR, holding breath) to the sound of the sniff (CS/trigger). With stimulus-response conditioning, a conditioned reflex can develop without a US/UR ( Donahoe and Vegas, 2004). A detailed explanation of stimulus-response conditioning is provided below.

**Figure 1.**
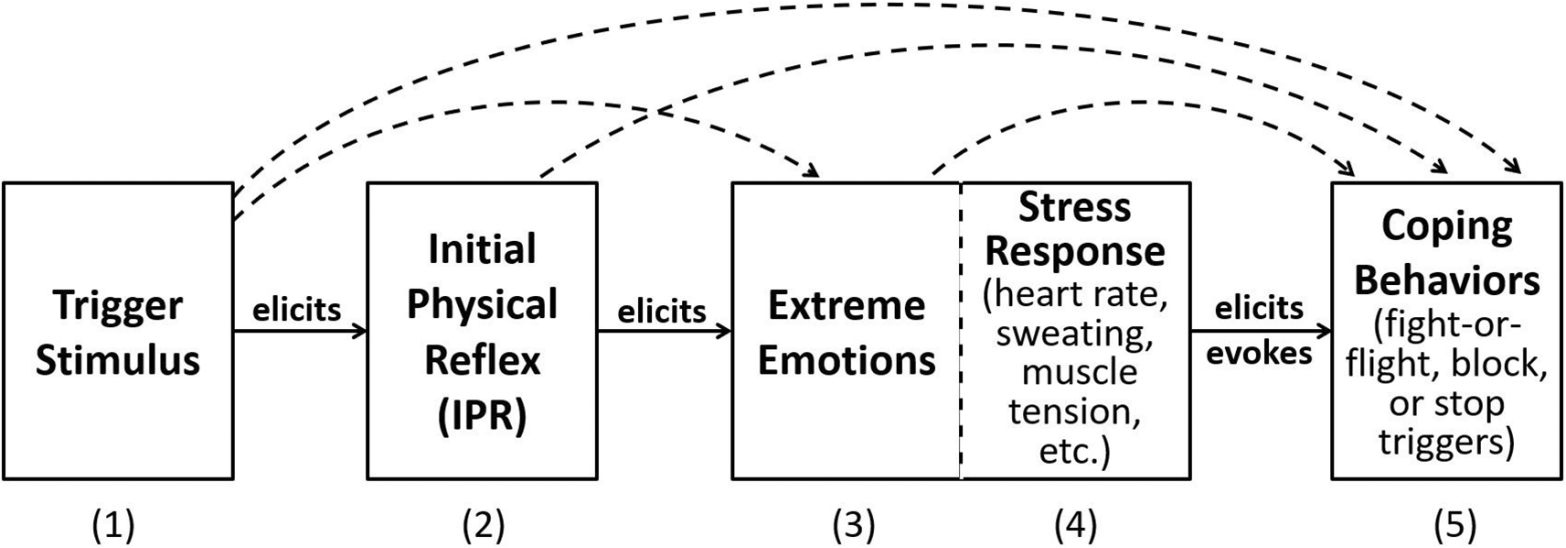
Dozier model of the hypothesized response chain of misophonia. The (1) misophonia trigger stimulus elicits a (2) physical (muscle) reflex response, which in turn elicits the (3) extreme emotional response, (4) stress response, and (5) overt behaviors. Dotted line connections indicate secondary effects.

The model states that, once developed, the IPR strengthens with *in vivo* exposure to trigger stimuli. Emotional responses then develop due to the intrusive and aversive nature of the muscle reflex. This behavioral model was expanded to include the physiological distress that accompanies strong emotions and overt coping behaviors ( Dozier, 2015d; Figure 1). In Figure 1, the trigger stimulus is perceived by the person and elicits the IPR. The internal sensation of the IPR in turn elicits the extreme emotions that are the hallmark of misophonia. Physiological responses elicited by intense emotions are shown as Stress Response. Finally, the experience of the extreme emotions and stress response elicit and evoke overt coping behaviors, which often consist of behaviors that reduce, terminate, or escape the trigger stimulus. Arched, dashed lines between the boxes indicate there are likely secondary, direct connections in addition to the primary response flow. In real life, the misophonic experience consists of repeating this linear model with each occurrence of the trigger, with increasing and co-occurring emotions, stress, and overt behavior. This model assumes the same mechanism is valid for auditory, visual, olfactory, and other sensory modality triggers. A strength of this model is that it is based on known neurological processes, namely Pavlovian conditioning and the development of learned emotional reflexive responses. Dozier presented brief cases that support Pavlovian conditioning of the IPR ( 2015b, 2017). Additionally, the model is supported by case studies that utilized this model ( Dozier, 2015a, 2015c, 2022) and studies investigating the existence of the IPR ( Dozier and Morrison, 2017; Dozier *et al.,* 2020), which are discussed below in detail.

There is much commonality between the Jastreboff and Jastreboff (2002) neurophysiological model, the Cowan *et al.* (2022) psychological model, and the Dozier (2015b) behavioral models of misophonia. The Jastreboff model elucidates connections among brain structures for misophonia. The Cowan model focuses on the interplay of psychological traits and responses which cause misophonia to develop. The Dozier model identifies a behavioral chain of events that occurs when a person perceives a trigger. These models essentially look at the topic of misophonia from three different worldviews. Unlike the Dozier model, the Jastreboff and Cowan models do not identify the etiology of misophonia as a Pavlovian conditioned physical reflex. In the Jastreboff and Cowan models, misophonia develops as an emotional reflex, but in the Dozier model misophonia develops as a physical (usually a skeletal muscle) reflex, and then the emotional reflex develops. For example, with the Jastreboff and Cowan models, a person hears a chewing sound in connection with something negative, allowing the conditioned emotional reflex to develop. But with the Dozier model the person hears a chewing sound and develops a Pavlovian conditioned physical reflex (usually a skeletal muscle response), and then the emotional reflex develops.

The current study presents the Mitchell-Dozier model, based on our observations in clinical work with misophonia patients and widely accepted cognitive-behavioral explanations of pathology development. Some aspects of this model are broadly recognized as characteristics of individuals with misophonia, but others are characteristics we see frequently, yet they remain underreported in the literature. Our goal is to clearly present this model, the components of which we see in patients on a daily basis. Our hope is that this model, along with plausible theoretical support, will provide novel predictions for misophonia treatment and a basis for directions of future misophonia research.

### Hypothesis and theory

**Figure 2.**
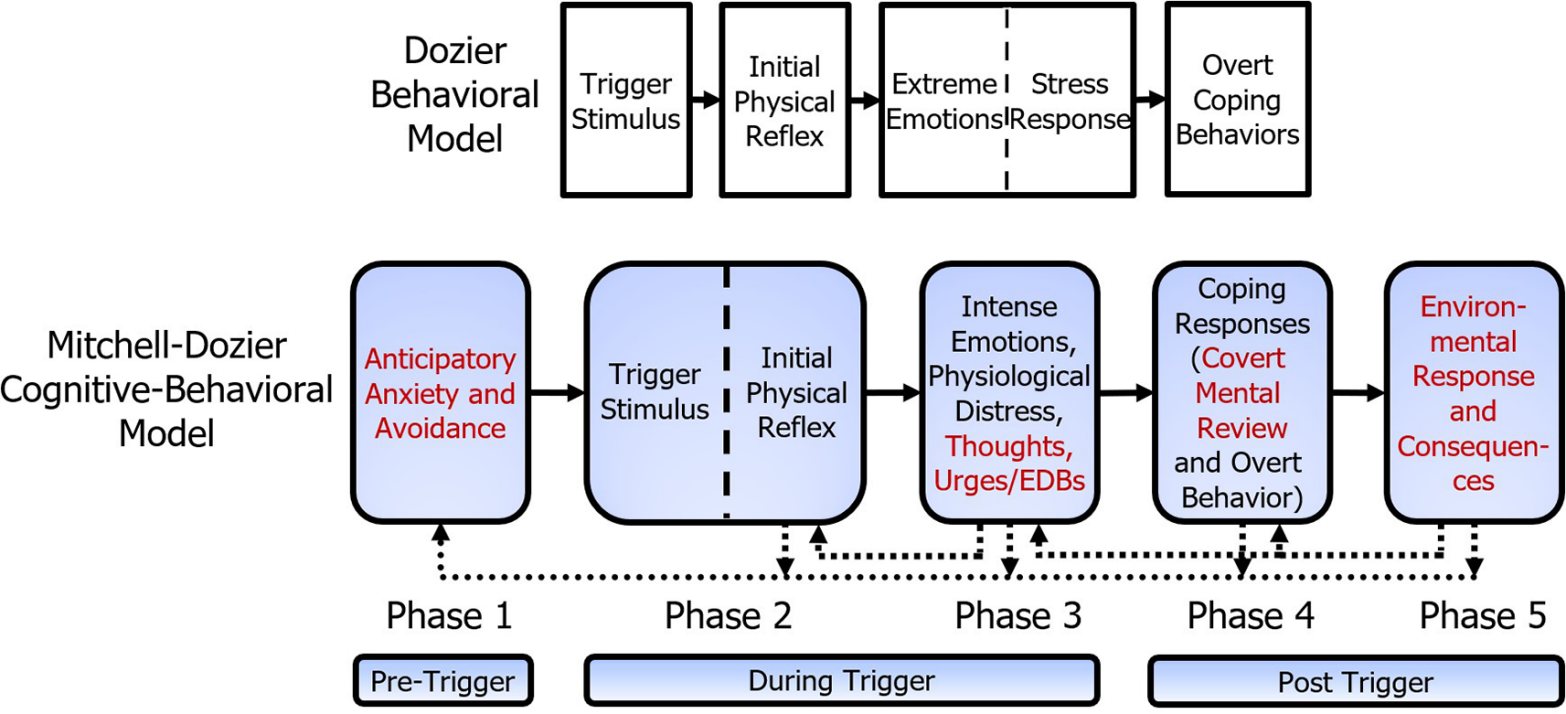
Comparison of Mitchell-Dozier cognitive behavioral model and Dozier behavioral model of misophonia. Additions to the Dozier behavioral model are shown in red.

The model presented in this section builds on the Dozier behavioral model ( Figure 1). Figure 2 shows the additional components of the Mitchell-Dozier model, compared to the Dozier model. Whereas the Dozier model is a behavioral model, the Mitchell-Dozier model is a cognitive-behavioral model. The additional components include anticipatory anxiety and avoidance (phase one), thoughts, urges, and emotion-driven behavior (part of phase three), covert mental review (part of phase four), and environmental response and consequences (phase five).

**Figure 3.**
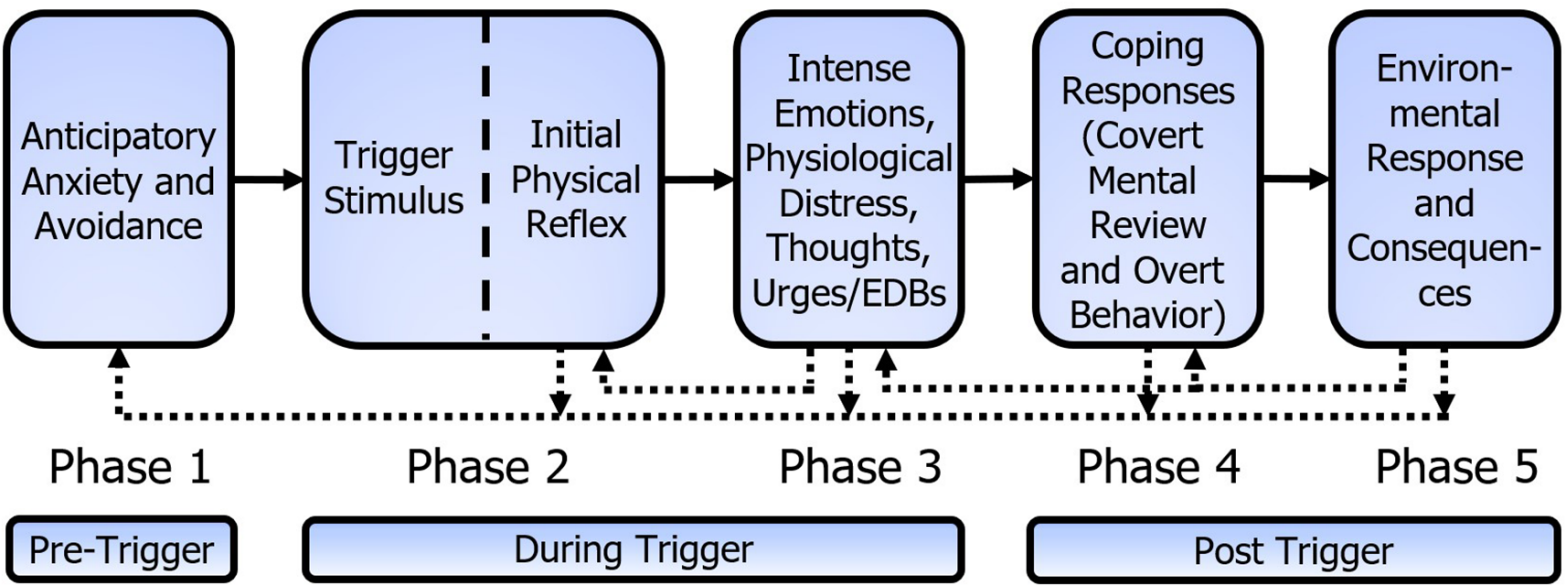
Mitchell-Dozier model of misophonia. (1) Anticipatory anxiety and avoidance, (2) the misophonic trigger stimulus elicits the initial physical reflex (e.g., muscle flinch), (3) intense emotional response of misophonia, physiological distress, thoughts, urges and emotion driven behaviors while the trigger continues, (4) coping responses after the trigger, including covert mental review and overt behavior, and (5) environmental response and internal and external consequences. The dotted connection indicates phase 3 contributes to strengthening of the initial physical reflex, phases 2 to 5 contribute to strengthening anticipatory anxiety and avoidance of phase 1 and phase 5 contributes to strengthening phases 3 and 4.

The Mitchell-Dozier model consists of five phases, as shown in Figure 3. At the core of the five-phase Mitchell-Dozier model is a conditioned physical reflex that develops through stimulus-response Pavlovian conditioning (phase two). In this case a physical reflex refers to an automatic (and therefore without cognitive consideration) physical response to a stimulus, such as skeletal muscle contractions (including breathing muscles), internal muscles (esophagus, stomach, intestine), or physical sensations such as pain, numbness, heat on skin, flash of warmth, etc. For example, the sound of chewing (CS) could elicit the tensing of calf muscles or holding one’s breath (CR). Phase three includes intense, negative, emotional responses such as anger, disgust, and anxiety, as well as accompanying physiological distress, thoughts, urges, and emotion-driven behavior. Phases two and three occur while experiencing the trigger and phase three ends shortly after termination of the trigger stimulus. Phase four is the individual’s post-trigger coping responses to the emotional and physiological distress, including covert mental reviews of the trigger and overt behavior. The fifth and final phase is the environmental response and resulting internal and external consequences of the individual’s coping behaviors. Phases four and five include the period from the termination of the trigger stimulus until the effects of the trigger experience extinguish. The repeated, aversive experiences of phases two to five lead to feelings of anticipatory anxiety and avoidance in the individual’s daily life; thus, these feelings and behaviors form the pre-trigger phase one.

Each phase attempts to explain the maintenance of the response and the individual’s subsequent impairment. Anticipatory anxiety and avoidance (phase one) contribute to increased arousal and greater awareness of triggers, resulting in an increased severity of the trigger experience. Phase two is a Pavlovian-conditioned physical reflex that strengthens with *in vivo* exposure to triggers. Phase three is a conditioned emotional response and associated physiological distress, thoughts, urges, and emotion-driven behavior, which also strengthens with *in vivo* exposure to triggers. Phase four includes both internal and external coping behaviors to the intense emotions and distress, in which patients regularly report a ‘mental review’ of the misophonic trigger as though it were an attack, which reinforces the belief that any subsequent trigger will be intolerable and must be avoided. Widely known are the aggressive or panic-like behavioral responses of individuals experiencing a misophonic trigger. Phase five includes internal and external consequences of misophonic individuals’ coping behaviors. Internal consequences include beliefs and new emotions based on environmental responses to anger and panic. These can include the development of emotions such as shame and guilt, beliefs regarding how ‘intolerable’ the trigger is, and intensified beliefs about the unfairness of having to experience the trigger. External consequences can serve as operant reinforcement for the explosive anger and/or panic behavior; for example, demands to stop the trigger and ‘death stares’ directed at the source of the trigger.

Taken together, phases two through five create overwhelming anticipatory anxiety and avoidant behavior (phase one) that perpetuate much of the dysfunction and impairment of misophonia. Phase one-related avoidant behaviors and emotions further intensify the learned responses associated with phases two through five, thus creating a dynamic, cyclical pattern of increasing dysfunction and despair. We present this as a linear model rather than the familiar circular model such as one for panic disorder ( Clark, 1986). With panic disorder, the individual may experience a cycle of increasing distress triggered by a single thought. With misophonia, it is likely that at a molar level, cyclical interactions and effects occur, but at a molecular level, phase one is a setting event for the severity of the misophonic response, which is fundamentally linear, initiated by each instance of the misophonic trigger stimulus. The sections that follow explain the phases in greater detail. We begin with phase one, but we emphasize that phase two is the vital component of Pavlovian conditioning of a physical reflex critical to the development of misophonia. We recognize that there are likely many predisposing factors, including emotional awareness of moods of others, sensory sensitivity, and genetics, but these are beyond the scope of this article.

## Methods

### Phase one – Anticipatory anxiety and avoidance

We believe the origins of misophonia begin with Pavlovian conditioning (phase two) and subsequent responses (phases three through five). However, once the misophonic trigger exists, patients often talk about the fear and avoidance of the trigger, which is one of the primary areas of dysfunction, as the first step in the process. Anticipatory anxiety and avoidance of triggers is commonly reported and seems to be a universal characteristic among misophonia sufferers ( Cusack *et al.,* 2018; Dibb *et al.,* 2021; Edelstein *et al.,* 2013; Jager *et al.,* 2020; Quek *et al.,* 2018; Schadegg *et al.,* 2021; Siepsiak *et al.,* 2020; Wu *et al.,* 2014). Avoidance of typical communal activities, such as eating together, enjoying social activities, and being in school or work environments, creates much of the dysfunction of misophonia. However, our model states that typical exposure to triggers increases the IPR (phase two) and intense emotions (phase three), therefore, simply eliminating avoidance in non-therapeutic environments is contraindicated. Anxiety related to experiencing triggers can increase physiological distress and hypervigilant behavior, such as scanning for sources of triggers (e.g., individuals walking into a classroom and scanning to see if anyone is chewing gum). Our clinical cases attest that this increases the salience of triggers, strength of the IPR, and emotional responses. Since the person is looking for or anticipating triggers, they are thinking about them, re-experiencing them, and thus are more likely to find them. Anxious anticipation may cause tensing of the IPR muscle and result in the actual IPR muscle response being perceived and objectively measured as more intense than would be the case otherwise. For a person who immediately holds their breath as their IPR, if their chest muscles are already tightened because of anticipatory anxiety, the physical sensation of the IPR (holding breath) is intensified. Furthermore, regarding intensified emotional responses, individuals who are triggered when already experiencing anxiety typically perceive and exhibit intensified emotional responses. Most individuals, whether they suffer from misophonia or not, can attest to experiencing emotions more intensely when already stressed.

Anticipatory anxiety can present differently between children and adults. We find that children often scan a particular family member for any indication of an increased likelihood of a trigger. For example, children may continuously scan a parent’s face to make sure their mouth is empty and chewing will not occur. This scanning behavior creates a dysfunctional environment, makes triggers more likely to be noticed and more severe, and can cause new triggers to develop to other physical actions. Per the previous example, the child becomes so intensely focused on possible chewing behavior that family functioning becomes difficult. The child may ask for reassurance, continually shift position to look at the face, and challenge the parent’s response that they don’t have anything in their mouth. The child may also have a strengthened or intensified response to the parent’s chewing behavior because of fight-or-flight responses prior to the trigger occurring. Further, as the child continuously observes the parent's facial expression, previously neutral events can become triggering stimuli; for example, a facial movement similar to chewing or something unrelated such as the parent adjusting their glasses.

### Phase two – The initial physical reflex

A largely understudied aspect of misophonia is the existence of an IPR, although it has been reported in several case and quasi-experimental studies. In one case, the patient reported experiencing tensing of face and shoulder muscles and a sharp pain in her sternum ( Dozier, 2015a). The pain in her chest was later ascribed to a reflex in which the IPR closed her windpipe and simultaneously caused her to inhale. Other case studies report an IPR of fist clenching ( Dozier, 2015c) and jaw clenching ( Dozier, 2022). Additionally, Dozier (2015b) reported IPR responses of seven individuals which included: shoulders; arms and chest; rightward head twist and right arm tense; arms, shoulders, and legs; goose bumps on upper arms; sensation behind the ears; and intestinal constriction. Therefore, phase two of the Mitchell-Dozier model is based on the limited data within some clinical case studies.

In a quasi-experimental study, 26 participants reported physical sensations in various locations when exposed to weak trigger stimuli ( Dozier and Morrison, 2017). Weak triggers and infrequent exposure to triggers ensured participants did not experience strong emotions or physiological distress. The locations, while consistent with each participant, varied between participants, with the location of physical sensations ranging from head to toe. Most of the reported physical sensations can be attributed to muscle tightening (e.g., jaw muscle, shoulder sensation), but non-skeletal-muscle responses included numb skin, flash of warmth, sexual sensation, and nausea. A key limitation of this study was the reliance on self-report data and the absence of any temporal measurements (specifically the trigger-to-response delay). A subsequent pilot study with only three subjects addressed these limitations through electromyography and direct observation ( Dozier *et al.,* 2020). Direct observation revealed a movement of muscle or tissue that seemingly occurred with no perceptible delay to trigger onset. The electromyography data indicated a trigger-to-response delay of approximately 200ms for auditory triggers and 350ms for visual triggers. Collectively, these reports provide some evidence of the existence of an immediate misophonia-related IPR in some individuals with misophonia.

Furthermore, two studies measured physiological arousal using skin conductance response (SCR) in response to triggers ( Edelstein *et al.,* 2013; Kumar *et al.,* 2017). In both studies, the SCR response was stable for the first two seconds of the trigger, and then rose monotonically for the remainder of the 15s trigger. However, SCR measures stress or physiological arousal at the skin level and cannot be used to measure immediate muscular flinches. In contrast to the delayed SCR response, the IPR begins within the first half-second following stimulus onset, so the delayed SCR response reported in these studies was not the IPR.

We propose that the IPR is different from the physical responses commonly reported in other studies. These studies reported physiological responses, including tense muscles, warmth, and increased heart rate, as physical responses to triggers. For example, studies report general muscle tensing, but an IPR would be specific muscles tensing, and be different from person to person. General warmth and increased heart rate are also physiological responses and not an IPR. Furthermore, numerous studies report that muscle tensing is often experienced by individuals when triggered in response to emotions (e.g., anger, anxiety), physiological distress, and fight-or-flight ( Dibb *et al.,* 2021; Edelstein *et al.,* 2013; Jager *et al.,* 2020; Jastreboff and Jastreboff, 2014; Potgieter *et al.,* 2019; Rouw and Erfanian, 2018; Schröder *et al.,* 2013; Schröder *et al.,* 2019; Wu *et al.,* 2014; Zhou *et al.,* 2017). Similarly, muscle tension and other physical responses reported by individuals as part of the misophonia response have generally been understood as part of the emotions and distress of misophonia. Within the Mitchell-Dozier model, however, these responses would be categorized as part of phase three (physiological distress), rather than the IPR of phase two.

It can be difficult to distinguish the IPR from other physical responses, but there are several ways to differentiate them. First, the IPR will be a consistent initial response for a specific trigger. For example, if a person’s IPR for a sniff trigger is shoulder tensing, then shoulder tensing will always occur when the person is triggered by a sniff. Also, the IPR will not contain topographic variations based on the current situation, such as orienting toward the person making the trigger. An orienting response would be part of phase three as a component of fight-or-flight or other defensive motivational responding. For example, one individual’s IPR was clenching his right arm and turning his head to the right. This response occurred every time he was triggered, regardless whether the trigger source was to the right, left, in front, or behind him. Further, because the IPR is a conditioned reflex, it will be elicited by the trigger stimulus and begin immediately (e.g., about 200ms) after onset of an auditory trigger ( Dozier *et al.,* 2020). For a single trigger (e.g., a sniff), any physical response occurring after the trigger stimulus stops is likely a defensive motivational system response, a physiological response to strong emotions, or other responses described in phases three and four. Finally, the IPR may be experienced without the emotional response or precursors of fight-or-flight when the trigger stimulus is very weak. Patients’ IPR responses reported by Dozier (2015b) were often experienced without emotional distress, and participants in another study often reported having a physical sensation but no emotional response or fight-or-flight precursors ( Dozier and Morrison, 2017), indicating that the physical response and the emotional response can be dissociated. Furthermore, another patient perceived tensing her jaw and fists to be her emotional/physiological response to triggers ( Dozier, 2022). However, with testing, she determined her IPR was jaw tensing, and clenching her fists and jaw was part of her general physiological arousal or anger response. Dozier and Morrison (2017) provide a procedure to determine the IPR. These cases demonstrate that, because it has been understudied, what individuals report as part of the emotional response (phase three) may very well include their IPR, and therefore a thorough investigation is required.

*Pavlovian conditioning and the initial physical reflex*

As stated above, we propose that the IPR seen in misophonia cases develops through Pavlovian conditioning. Dozier (2015b) described four cases where the physical behavior (muscle tensing) that occurred when misophonia developed was the same as the muscles of the IPR. For example, a child who, during mealtime arguments, would stand and angrily extend both arms toward her brother. Her IPR for eating triggers was in her quadriceps, shoulders, and upper arms, which were the muscles used to stand and extend her arms. The aforementioned quasi-experimental study in which 26 individuals identified their physical sensations reported that the responses varied greatly between individuals ( Dozier and Morrison, 2017). Although there may be multiple explanatory factors, this variation could indicate that responses are learned rather than developed through a common neurological abnormality, thus supporting Pavlovian conditioning as the etiology of these muscle responses to commonly occurring stimuli. Further support is provided by reports of individuals who developed muscle responses to a pager ( Dozier *et al.,* 2020), chemotherapy pump ( Dozier and Morrison, 2017), and phone ring tone ( Dozier, 2015b). One individual reported developing a new trigger to a sound that was paired with a trigger during an experimental treatment, where the initially neutral stimulus (a ‘ping’ sound) elicited the same IPR as the trigger ( Dozier, 2015b). Additionally, spontaneous recovery has been reported in connection to treatments that reduced the IPR ( Dozier, 2015b). Given that spontaneous recovery is a well-documented characteristic of conditioned reflexes ( Catania, 2013), these findings support Pavlovian conditioning as the origin of the IPR ( Dozier, 2015a, 2015b, 2015c, 2022).

Sometimes we understand the development of the IPR as a response to a truly US (e.g., yelling at a meal) causing a startle response (UR) which is paired with the sound of chewing, so that chewing sound becomes the CS, and the startle response becomes the CR (IPR). Furthermore, we also see potentially second-order classical conditioning examples, where emotional responses develop through recurrent conditioning events. We argue that emotional responses (e.g., concern, offense, worry) often include individual unconscious physical responses (e.g., one person may raise their shoulders, while another person tightens leg muscles). The unconscious physical component can then become associated over time with the sounds of a parent, sibling, or partner, so that the sound becomes a CS that elicits the CR (IPR). For example, a child may acquire a reflexive or consistent unconscious muscle response to a parent’s scowl (e.g., they hold their breath). If the parent scowls when hearing a chewing trigger, then the child will hold their breath and could develop a hold-breath reflex response (IPR) to that sound. We emphasize that this is a conditioned physical reflex (i.e., muscle tensing) and not a conditioned emotional reflex. However, we propose that a conditioned emotional reflex does develop, and this is described in phase three.

For the purpose of this manuscript, a response is any form of behavior. A response can either be a reflex response elicited by a specific stimulus or an operant response evoked by a stimulus and learning history (i.e., operant conditioning). A conditioned (learned) reflex response occurs immediately when the stimulus is perceived, without thought. A person can choose to not emit an operant response but has no willful control to prevent a reflex response. Emotions are reflexive behavior which can occur due to conditioning with a specific stimulus, or can occur due to a thoughtful, evaluative process. In this manuscript, we refer to conditioned emotional responses as those which develop and are emitted immediately upon experiencing the stimulus. Our distinction between physical and emotional reflex responses is critical. A physical reflex is a purely physical action of the body. For the IPR of this model, it is usually a skeletal muscle flinch, but it could be something else (e.g., pain sensation, sexual sensation, or goosebumps). A conditioned emotional reflex is an emotional response elicited by a stimulus with accompanying physiological responses. We always refer to physical responses from emotions as physiological responses, not physical reflexes. We consider the IPR a conditioned physical reflex and the emotional responses of phase three conditioned emotional reflexes, with accompanying physiological responses. Phase three also includes evoked operant behavior (e.g., screaming) and elicited reflexes (e.g., fight, fight, or freeze).

*Stimulus-response Pavlovian conditioning*

The typical understanding of Pavlovian conditioning is that learning takes place when a neutral stimulus (NS/CS) is paired with an US/UR, or for second-order conditioning, an established CS/CR, whereby acquisition of the CR is driven by the CS-US pairing ( Ghirlanda and Enquist, 2019). However, with misophonia, there is usually no clear US/UR, and there have been no examples of US/UR reflexes reported in the misophonia literature. Interestingly, an alternative understanding of classical conditioning emerged in the mid-2000s, known as stimulus-response (S-R) Pavlovian conditioning, which has direct application to misophonia. S-R Pavlovian conditioning proposes that a response develops due to an association between the NS/CS and the physical response. For example, Donahoe and Vegas (2004) reported an S-R association in the muscle response of pigeons swallowing. The stimulus-to-response delay from water-into-mouth (US) to swallowing (UR) was a half-second. With this delay, the researchers tested forward and backward conditioning of the CS to the US (water-in-mouth) and to the UR (swallowing). Forward conditioning (CS before both US and UR) reliably developed a condition response, while backward conditioning (CS after both the US and UR), as expected, did not. However, backward-to-US and forward-to-UR (CS after the US and before the UR) reliably developed a CR. The study concludes that it is the CS-UR, or S-R, relationship that is critical to the development of a conditioned muscle response. Therefore, a US/UR reflex is not required. All that is required is a consistent pairing of a stimulus and a response.

The S-R view of Pavlovian conditioning is consistent with the neurology theory that the brain attempts to minimize expectation error by predicting responses based on prior experience, and thus tensing the muscle following the stimulus ( Haruno and Kawato, 2006; McClure *et al.,* 2003). In S-R Pavlovian conditioning, when a stimulus is predictably followed by a muscle response the stimulus becomes a CS and the muscle tensing becomes the CR. Note that the muscle tensing response must predictably follow the stimulus, but an US is not required to develop a S-R Pavlovian-conditioned reflex. Unfortunately, to date there are no published studies of S-R Pavlovian conditioning, either in animals or humans; nor are there any studies confirming or refuting the findings of Donahoe and Vegas (2004). This is clearly a research opportunity, so we only have case data for S-R conditioning in humans.

For example, in a case referred to above, a trauma center surgeon developed a shoulder flinch response to his pager ( Dozier *et al.,* 2020). The surgeon stated that when the pager initially sounded, he felt a sense of dread about what gruesome injury he might face in the trauma center. With the dread emotion, there was a slight physical response (i.e., shoulder tensing). With repeated pairing the CR developed and strengthened into a shoulder muscle jerk, and every time his pager went off (CS), his shoulders flinched (CR), until he changed jobs and stopped carrying a pager.

S-R Pavlovian conditioning of a muscle response has direct application to our assertion of how misophonia develops and why the misophonic response strengthens with typical exposure to trigger stimuli. We begin with an explanation of how the IPR develops. When a particular person perceives a common stimulus (e.g., chewing sound) and consistently has a thought with an accompanying specific muscle tensing (e.g., biceps), the chewing sound becomes a CS/trigger that elicits the tensing of the biceps (CR/IPR). For example, a person could hold their breath when hearing eating sounds they deem inappropriate. This results in a temporal pairing of eating sounds and breath holding. The eating sounds would become the CS/trigger and breath holding would be the CR/IPR. This could describe the development of a person’s first misophonia trigger or a subsequent trigger that is unrelated to previously developed triggers. The stimulus and the response are likely mediated by a negative thought or emotion. Patients report varied situations in this regard. Possible scenarios include an unconscious behavior unrelated to the stimulus, such as the child struggling to breathe due to asthma ( Dozier 2015a); annoyance, such as sound emanating from a neighbor’s property ( Ferrer-Torres and Giménez-Llort, 2021); someone breaking etiquette rules, such as chewing loudly; or a sense of unfairness, such as being criticized for chewing loudly, but then hearing a sibling chewing. Indeed, there are numerous possibilities for a sound and negative thought to result in an unconscious muscle response that could enable the development of a Pavlovian-conditioned muscle response. Thus, the natural occurrence of a stimulus, followed by a specific muscle response, can easily develop a trigger/CS with a CR/IPR of that specific muscle.

Individual differences in perception of events and responsiveness to stimuli may cause one person to respond to a stimulus when others do not. Wu *et al.* (2014) reported a strong association between misophonia impairment and general sensory sensitivity, and moderate associations with anxiety, depression, and obsessive-compulsive symptoms. Additionally, individuals with anxiety display an attentional bias towards threatening stimuli ( Cisler and Oster, 2010). Similarly, Fox *et al.* (2002) reported a correlation between heightened trait anxiety and increased attention to emotional facial stimuli. Thus, individual differences in awareness and responsiveness to common stimuli and family dynamics can cause one child to develop a misophonic trigger in response to common sounds but not their sibling.

*Strengthening of the initial physical reflex*

We will next consider how misophonia triggers can strengthen. We propose that the IPR strengthens with typical *in vivo* exposure to triggers. With S-R conditioning, once the ANS has developed the CS/CR reflex, every experience of the CS (which includes context) creates an S-R conditioning learning event. When the CS is detected, the CR is elicited. If there is no additional response in the CR muscle, it creates an extinguishing event. If there is slight additional tensing in the CR muscle, it creates a reflex maintenance event, and if there is considerable additional tensing in the CR muscle, it creates a reflex strengthening event. Note that the traditional view of reinforcement of a conditioned response has questionable application for S-R conditioning. With S-R conditioning, the theory is that the brain tries to reproduce the CS/CR reflex to match prior behavior. As the physical response is intrusive, the person may tighten the muscle of the IPR after experiencing the reflex and so it maintains or strengthens the reflex. The intense emotions of misophonia (phase three) have accompanying physical responses which may include tightening the muscle of the IPR. This is shown in Figure 3 as the feedback connection from phase three to the IPR. A trigger thus creates a conditioning event because the muscle response after the reflex is stronger than the reflex response alone. This proposition is consistent with the report that mere exposure to misophonic triggers generally increases misophonia symptoms rather than reducing them ( Schröder *et al.,* 2017). We therefore posit that typical exposure to misophonic triggers maintains or strengthens the IPR.

*Expansion of trigger stimuli*

Once misophonia has developed in an individual, evidence suggests that new triggers can develop in three ways: 1) independently of existing triggers, 2) by pairing with an existing trigger, or 3) though generalization of trigger stimuli. The same process that allows the first misophonia trigger to develop can allow the development of other triggers, independent of the first trigger. When a second trigger develops independently, it may have a different IPR than the first trigger, or it may have the same IPR. One study reported that 42% of the 26 people tested for their IPR had distinctly different IPRs for different triggers ( Dozier and Morrison, 2017). For example, one individual reported an IPR of shoulders tensing in response to the sound of someone chewing and an IPR of fist clenching in response to the sound of someone sniffing. However, note that IPRs are not necessarily different for each trigger. A person may have a characteristic response to a stimulus they perceive as intrusive (e.g., tensing neck muscles), and therefore a new trigger that develops independently of other triggers may have the same IPR as one or many other triggers.

When a NS (new CS) is paired with an existing trigger (CS), it is also temporally paired with the existing trigger response (CR/IPR), so the new CS has the same CR/IPR as the established trigger. For example, it is common for misophonic individuals with auditory eating triggers to develop a visual trigger of jaw movement (i.e., chewing; Claiborn *et al.,* 2020; Dibb *et al.,* 2021; Edelstein *et al.*, 2013; Jaswal *et al.,* 2021; Swedo *et al.,* 2022). By noticing jaw movement while being triggered by the sound of chewing, jaw movement becomes a CS that elicits the same CR/IPR as the sound of chewing. Once the visual trigger develops, it can maintain or strengthen even without continued exposure to the auditory trigger. An example is the woman who had auditory and visual triggers of her husband scratching his beard ( Dozier, 2015a). The auditory trigger was greatly reduced through counterconditioning, but the visual trigger remained unchanged.

Finally, trigger stimuli will generalize to other settings, sources, and variations of the sound of the original trigger stimulus. Once a trigger generalizes to a new environment, source, or variation, the response will strengthen with continued exposure to the trigger. We argue that all three ways of developing new triggers are the result of natural S-R Pavlovian conditioning events that occur in typical daily life. As such, the perceived experience of misophonia is often one of ever-growing distress and dysfunction and seems to rarely subside without proper treatment.

We reiterate that while the IPR is overwhelmingly a skeletal muscle response, it can be virtually any muscle or physical response. Less common responses could be a sexual sensation, sensation of warmth, nausea, or numbness of skin ( Dozier and Morrison, 2017). Additionally, our clinical experience includes IPR of pain at a specific location, sensation of burning on the skin, an itch, goose bumps, intestinal contractions, stomach contractions, and urge to urinate.

### Phase three – Intense emotional response

The third phase of the model is ‘intense emotional response’. At first glance, it may seem as though this aspect of misophonia is the most understood. Indeed, intense emotional responses are the primary complaint of both individuals who suffer from misophonia and their loved ones. The variety of emotional responses has been widely documented. There is little debate in the literature as to the many emotional experiences that overwhelm and plague individuals with misophonia. Dozier (2015b) proposed that the negative emotions develop because of the aversive and intrusive nature of the IPR. Aversive stimuli have been shown to elicit negative emotional responses. Research using rats showed an immediate aggressive response is elicited by electrical shock ( Ulrich and Azrin, 1962), while research on humans indicates aversive stimuli evoke fight-or-flight emotions and overt behavior ( Berkowitz *et al.,* 1981; Berkowitz, 1983). Furthermore, aversive olfactory and gustatory stimuli were reported to elicit activation of the limbic system ( Zald and Pardo, 1997; Zald *et al.,* 1998). These studies provide plausible support for the assertion that the aversive nature of the IPR (phase two) contributes to the development and maintenance of the emotions of misophonia; however, once the IPR develops, there are many, varied emotional experiences that occur in the context of each trigger event.

Additionally, brain imaging research indicates the emotional response is a reflexive learned emotional response ( Kumar, 2015; Kumar *et al.,* 2017; Schröder *et al.,* 2019), though no information on the temporal relationship of the emotional response and IPR is provided due to the three-second sample rate of fMRI. The anterior insula cortex, which is central to the misophonic emotional response, integrates interoceptive and exteroceptive stimuli, so the interoceptive sensation of the IPR would be included in the overall sensory experience which triggers the emotional response. The specific brain structures involved are described in the introduction, but do not include any of the brain structures identified for Pavlovian conditioning of a muscle response ( Thompson, 1990). Therefore, we posit the learning of the emotional response of misophonia is separate from the learning of the IPR. Instead, the reflexive emotional response develops due to the repeated experience of the intrusive and aversive IPR, along with simultaneous emotional experiences (e.g., conflict with the person producing the trigger or frustration from lack of control).

Though there is no published research on S-R conditioning and development of emotional responses in humans, there are three published misophonia cases where the individual experienced a weak physical response (IPR) but no emotional response with exposure to weak trigger stimuli during treatment ( Dozier 2015a, 2015b, 2015c). This supports the distinct separation of the IPR and the emotional response. In the Mitchell-Dozier model, the trigger stimulus elicits the IPR (phase two), and the IPR elicits the emotional response (phase three).

The purpose of this section is not to provide new information regarding the intense emotional responses. Rather, we provide a cognitive-behavioral structure to understand these emotional responses, a structure which is linked to current understanding in the field at large, as well as to empirically supported treatments for other commonly comorbid disorders.

In our model we attempt to explain emotional responses from the tripartite model of emotions presented in the Unified Protocol for Transdiagnostic Treatment of Emotional Disorders ( Barlow *et al.,* 2018). This model presents emotions as having physical, cognitive, and behavioral components to responses (see Figure 2). From this model, we assert that the intense emotional responses reported by patients will have three vital aspects. First, we attempt to understand the intense physiological discomfort. It is important to note that while the physiological discomfort is separate from the IPR, it can present similarly. Second, we attempt to understand the unhealthy, automatic thoughts experienced by patients. Third, the intense emotional responses must also include an exploration of the strong behavioral urges to avoid the discomfort. Below, we provide a brief explanation of each of these three core components to emotional responses.

During an intense emotional response, patients will experience physiological discomfort at a level tantamount to a physical attack. Patients often report feeling as though the misophonic trigger caused pain in some capacity. The IPR of phase two is very strong in some patients. One woman described her experience of being triggered as feeling as though a “shovel was run through her sternum and out her back” ( Dozier, 2015a). Many patients have a strong muscle reflex that feels like receiving an electrical shock ( Dozier, 2015c, 2022). The IPR occurs with each instance of a trigger stimulus and continues for the duration of the trigger, so for a longer trigger (e.g., a single long snore), the IPR triggers phase three and both phases continue until the trigger stimulus stops. Therefore, the misophonia sufferer experiences both phase two and phase three simultaneously, often with greater intensity due to the individual’s reflexive response to the sensation of the IPR. Additionally, there is an unconditioned physiological distress response to the intense emotions. Other commonly reported physical responses which occur in addition to the IPR include increased heart rate, sweating, general muscle tension, and tension of specific muscles associated with anger and distress (e.g., jaw and fist clenching). The physiological discomfort cannot be emphasized enough. We have found physiological discomfort is often the primary complaint of the patient during clinician assessments. However, because the intense emotional response is not always expressed behaviorally as ‘painful’, this aspect is often underappreciated by clinicians. Patients frequently report typical ‘fight, flight, or freeze’ responses, indicating the brain perceives the trigger as threatening.

Next, it is vital to explore the unhelpful automatic thinking associated with the intense emotional responses of misophonia. Patients frequently report unhelpful thinking, such as very frequently perceiving the trigger as an ‘assault’ or an affront. Social media was used to identify thoughts in response to triggers including thinking that it is on purpose to irritate the individual, disgust, hyper-fixation on the sound, and even threats or violent thoughts. As such, it is understandable when patients frequently report specific thoughts regarding the unfairness of the trigger, worries about the next possible trigger, and beliefs about the intolerance of their discomfort. Further, from numerous patient encounters and interviews, we now understand it is an incredibly common occurrence for patients to ‘mentally review’ specific qualities of the trigger. While no research has examined this phenomenon in detail, we believe given the perception of the triggers being an ‘attack’, it makes sense that the brain would remember details of ‘the attacker’ (i.e., the sound). Further, the perception of the triggering event as an attack helps makes sense of the ‘hypervigilance’ patients naturally exhibit after the triggering event.

Finally, from this model, emotions are also understood to ignite an automatic motivation, known as ‘action tendencies’, to engage in some behavior related to the strong physiological and thinking responses. Action tendencies generally result in “emotion-driven behaviors” (EDBs, Carver and Harmon-Jones, 2009; Christensen *et al.,* 2019; Lewon and Hayes, 2014). The behavior feels out of control, automatic, and incredibly difficult to resist. Examples of EDBs include orienting responses, negative facial responses, verbal demands, or physical aggression ( Bossuyt *et al.,* 2014). The application of EDBs to misophonia seems very clear on the surface as there are numerous reports of explosive externalizing, destructive behaviors. However, when a patient with misophonia is triggered, a wide array of emotions can be elicited, and thus, a wide array of both external and internal action tendencies occurs.

Avoidance and approach are action tendencies commonly reported as instinctive responses to anger and fear, respectively, although they can both be elicited by various emotional states ( Bossuyt *et al.*, 2014; Klein *et al.*, 2011; Sawashima, 2019). Disgust, however, an emotion commonly reported by individuals with misophonia, has the dominant action tendency of behavioral avoidance ( Izard, 1993). The observed avoidance can include both active and passive features, both of which can be viewed in individuals with misophonia. Active avoidance involves terminating exposure to the stimulus by moving away from the perceived trigger or shouting ‘stop’ at the individual performing the triggering behavior. Passive avoidance involves actions like closing the eyes or covering the ears. Approach and avoidance in various forms seem to be common action tendencies in misophonia.

With our patients, we discuss how behaviors/choices are seemingly automatic, but we try to emphasize that it is the ‘action tendencies’ or urges that are automatic, rather than the often-regrettable behaviors/choices. Doing so allows the patient to have hope that the EDBs are indeed modifiable and that the urges can eventually be resisted through treatment. Although these thoughts, urges, and EDBs occur as part of the intense emotions of phase three, they generally continue, along with the physiological distress, and become part of the coping behavior of phase four.

### Phase four – Coping responses

Phase four is non-reflexive, operant behavior that occurs after the trigger stimulus has stopped plus residue of the behavior of phase three (e.g., emotions, physiological responses, and EDBs), and automatic behavior provoked by cognitions and actions after the trigger stimulus has terminated. It can be the time after a single trigger (e.g., a single sniff), the time between intermittent triggers, or the time after a series of triggers has ended (e.g., person leaves the room or puts on headphones to block triggering sound). Phase four is primarily the individual’s coping responses to intense emotional and physiological distress, including both internal (covert) and external (overt) coping behaviors. The duration of this phase can vary greatly between individuals, with one validation study of a misophonia instrument reporting the time to recover from a trigger experience and ‘feel normal’ varies from almost immediately to longer than 24 hours ( Dibb *et al.,* 2021). A key distinction for phase four is that the trigger stimulus has ceased, so the IPR and the learned emotional responses elicited by the trigger experience have ceased. However, the physiological distress and intense emotions do not instantly cease, thus they continue into phase four.

Internal coping strategies are primarily a mental review of the specific nature of the trigger, anger rumination, and thoughts associated with the many possible misophonia emotions, including ways to escape the trigger. Patients regularly report a prolonged mental review of the misophonic trigger as though it were an attack, which reinforces the belief that the next trigger will be intolerable and must be avoided, thus strengthening the anxiety and avoidant behaviors of phase one. Research suggests that anger rumination, which has been identified as a component of other disorders (e.g., OCD), is associated with trait anxiety/negative affect rather than a specific disorder ( Jessup *et al.,* 2019). We find that the mental review of misophonia triggers often includes anger rumination. The mental review also prolongs the individual’s focus on the trigger and its source. This maintains or increases salience and responsiveness to subsequent trigger stimuli, likely strengthening phases two and three. The mental review of the trigger can cause the person to retrigger themselves, prolonging the distress and strengthening the urge to engage in hypervigilant behavior.

Widely known are the aggressive or panic-like behaviors commonly reported by individuals with misophonia. One such behavior is mimicking of oral triggers sounds, for which a neurological basis has been proposed ( Kumar *et al.,* 2021). Additionally, as a method of eliminating the trigger or communicating to others in the environment that they are upset, patients commonly report they emit facial and bodily responses (e.g., death stares). Patients often become aggressive and ‘verbally snap’ or ‘verbally assault’ the person making the trigger. In some cases, patients, usually children, become physically violent. While these overt behaviors generally succeed in stopping the triggering behavior, they can also create undesired consequences.

### Phase five – Environmental response and consequences

Phase five includes the internal and external consequences that result from coping behaviors. Internal consequences include beliefs and new emotions based upon environmental responses to anger and panic; for example, the development of emotions such as shame and guilt, and beliefs regarding how ‘intolerable’ the trigger is. Further, explosive anger or panic can produce consequences from others; namely, they try to stop the trigger.

Parents of children with misophonia can unintentionally reward explosive behavior by terminating the trigger; however, parents often blame the child for being ‘out of control’ or being ‘dramatic’. Parents and partners of individuals with misophonia report ‘walking on eggshells’ or ‘being controlled’ by the misophonic individual. But behaviorally, if screaming makes the pain stop, the individual is going to keep screaming. As such, these reactions from others, which seemingly accuse the patient of a deficit of character, are actually maintaining or increasing the explosive behavior. These environmental consequences shape the operant behavior in phase four and, though much of the behavior in phase three is reflexive or automatic in nature, consequences shape the operant behavior of phase three. This effect is shown in Figure 3 with a dotted line from phase five to phases four and three.

We often find environmental changes are necessary in order to decrease explosive behavior. Parents and loved ones must be trained to not engage in rescuing behaviors which inadvertently reward the misophonia sufferer’s explosive attempts at stopping the trigger. Further, we find that training parents and loved ones to properly view misophonia as an illness can help to decrease responses which cause guilt and shame in the sufferer.

Environmental changes (putting in ear plugs or moving to another location) also function as a reinforcer because they allow the patient to escape the trigger stimulus. The reduction in exposure to the trigger is a reinforcing consequence for the overt behaviors and so the patient continues to emit these behaviors. Unfortunately, due to the prolonged duration of phase four, simply stopping the trigger may not alleviate the negative effects from being triggered, which can persist for an extended period of time, sometimes even continuing into the next day.

The misophonic response to triggers is a repeating cycle, whereby each phase strengthens both the previous and subsequent phase. As noted earlier, phases two to five contribute to the creation of an aversive emotional and physical experience, and can increase the sufferer's belief that the trigger experience is simply intolerable. This view of the misophonic experience as being intolerable increases hypervigilance, anticipatory anxiety, and avoidance, which is phase one of the Mitchell-Dozier model.

## Discussion

We present a theoretical model of the development of pathology in misophonia to inform individuals, clinicians, and researchers in their implementation of interventions and research studies. We argue that the development of a pathology should be inextricably linked to treatment interventions. Furthermore, we want to be able to help patients understand why their pathology has developed and therefore why the chosen interventions are reasonable, understandable, and likely to produce benefit, including symptom reduction. Much like understanding the pathology development in social anxiety vs. panic disorder vs. phobias, the understanding of how misophonia is developed and maintained is vital to proper treatment. For every single disorder, there are researchers who have created sub-CBT models of pathology development to predict treatment.

Much of this model is likely viewed as common knowledge for those familiar with misophonia, so to emphasize the unique and critical features of this model, we repeat them here. The unique and critical features of this model are the IPR and mental review of phase four. The IPR has not been acknowledged by misophonia researchers, and it is generally not acknowledged by individuals with misophonia. However, within our clinical setting we find that virtually all of our patients acknowledge the IPR after simple tests are conducted in a treatment session. The other unique phase of this model, covert mental review, is often acknowledged, but the importance of this feature of misophonia in reducing misophonia severity is not appreciated. We find that reducing covert mental review of triggers can reduce the dysfunction of misophonia. Therefore, the unique and critical features of this model have found much support within the clinical setting ( Dozier, 2015a, 2015c, 2022); however, more robust research is required to confirm these findings on a larger scale.

This Mitchell-Dozier model of misophonia provides a coherent theory of misophonia as a condition which develops initially as a Pavlovian-conditioned physical reflex (phase two, muscle reflex), and subsequent conditioned emotional responses (phase three). We posit that through Pavlovian conditioning, the misophonic trigger can be any sensory experience and can be created by any environmental source. Some have proposed that misophonia be limited to stimuli of particular sensory modalities and require specific types of triggers (i.e., mouth and/or nasal sounds) to occur in order to be considered misophonia ( Schröder *et al.,* 2013; Jager *et al.,* 2020). We believe this definition is too narrow. While approximately 96% of misophonia sufferers have mouth-sound triggers and 85% have breathing/nasal-sound triggers ( Claiborn *et al.,* 2020; Jager *et al.,* 2020; Rouw and Erfanian, 2018), there are individuals who have neither of these. We do not know of any Pavlovian conditioning research that restricts the sensory domain of a conditioned stimulus, and our work with patients provides examples of auditory, visual, olfactory, tactile, and vibration trigger stimuli. We therefore propose that misophonia is present when a person develops consistently intense negative emotions and/or dysfunction from any specific innocuous stimuli. For example, one patient did not have any oral or nasal triggers and therefore did not meet the criteria of misophonia per Jager *et al.* (2020), but she had severe distress and dysfunction from non-oral/nasal innocuous stimuli (e.g., sound of pouring dry dog food into a metal bowl). The patient exhibited a breath-holding reflex (phase two) regardless of volume. Therefore, even though the sounds were atypical for misophonia, we view the patient’s condition as misophonia. The existence of misophonia was further supported by the patient’s positive response to behavioral treatment (e.g., counterconditioning). As her conditioned physical response extinguished, all other phases of the model extinguished. We propose misophonia be defined based on the conditioned response of the individual rather than requiring specific stimuli that trigger the response. As such, we assert misophonia can be triggered by any innocuous stimuli across all sensory modalities.

Outside of the phases of the Mitchell-Dozier model, we understand there are predisposing or vulnerability factors, some of which are known and some of which are not. For example, an association between general sensory sensitivities, (sensory over-responsivity) and misophonia has been reported ( Wu *et al.,* 2014). It is important to note that we believe misophonia is not an example of sensory over-responsivity but rather a conditioned response. Additionally, an association between anxiety and misophonia severity has been reported ( Cusack *et al.,* 2018; Quek *et al.,* 2018; Schadegg *et al.,* 2021; Wu *et al.,* 2014). The predispositioning factors of anxiety are numerous and well documented. The second author utilized the current understanding of panic disorder as a means to better understand misophonia. For example, interoceptive awareness seems to be a commonly reported experience in his misophonia treatment practice. More specifically, individuals who are highly ‘attuned’ to their physiological changes may be more likely to develop a Pavlovian response to stimuli that cause a physiological change.

We understand there are neurological pathways and brain functions that are presently not fully known; however, there is a good deal of evidence supporting a neurological basis of misophonia. As with all mental health issues, there is likely a biological contribution to misophonia. Additionally, there is a genetic contribution, as reported by 23andMe DNA testing ( Fayzullina *et al.,* 2015). Environmental contributions are vital to the development of misophonia given that it is a conditioned/learned response. However, the contribution of all the social/cultural/demographic variables to the development and maintenance of the disorder remain unknown. Additionally, reported age-of-onset data indicate misophonia can develop at any age ( Claiborn *et al.,* 2020). There may be social disparities and discrimination which increase conditioned aversive responses and therefore may contribute to the onset of misophonia; however, there are no data on this, so it is an opportunity for future research. Taken together, we understand there are numerous and varied bio-psycho-social factors which may increase or decrease the probability of developing misophonia.

### Clinical implications

We assert that misophonia is a chronic illness which must be continually managed, but we do not propose a cure. We view misophonia as a result of a normally functioning neurological process (i.e., Pavlovian conditioning). Because misophonia is a conditioned response, even when all symptoms extinguish, the patient remains vulnerable to developing new triggers or the reemergence of prior triggers. A brain imaging study ( Kumar *et al.,* 2017) reported that, compared to control participants, misophonia individuals had higher activation in brain areas responsible for emotional responses when exposed to unpleasant sounds, possibly indicating that a person with misophonia is more responsive to sounds deemed unpleasant and therefore more likely to develop a misophonia trigger. CBT for misophonia emphasizes the importance of not only extinguishing triggers, but continuous practice of techniques as well as altering vulnerability factors when and where possible. Further, the importance of meaningfulness and quality of life is utilized to cope with accepting the reality of a chronic illness and providing motivation for consistent management.

As with other CBT theoretical models of pathology development, the Mitchell-Dozier model is directly linked to misophonia treatment, presently called “cognitive-behavioral therapy, misophonia” (CBT-M, second author) and “relaxation and counterconditioning therapy” (RCT, Dozier, 2022). Utilizing the Mitchell-Dozier model, CBT-M and RCT treatments have shown promising results in our practices, though there is a lack of scientific study of these treatments. Both therapies incorporate intensive progressive muscle relaxation training and counterconditioning. This model elucidates two important aspects of the nature and features of misophonia which are poorly understood implications for treatment: the Pavlovian response of phase two and the mental review of phase four. Within clinical settings, many patients report significant reduction in misophonia severity when the Pavlovian response is treated directly through behavioral techniques as reported in three case studies ( Dozier, 2015a, 2015c, 2022). Additionally, patients often report increased quality of life by learning to address mental review, which is a newly identified feature of misophonia observed within our clinical practice.

### Alternative explanations

It is important to acknowledge that additional independent research is needed to validate this model and the effects of each phase as discussed above. It is possible that the phases of misophonia shown in this model may not have the causal effects we have ascribed to them. For example, the IPR may not be present in all misophonia cases. Furthermore, if the IPR does exist in all cases, it may simply develop as a result of repeated negative emotional responses to the trigger (i.e., phase 3 develops first, then phase 2). Alternatively, the IPR may occur simultaneously with the emotional response rather than preceding and eliciting the emotional response, or add to the overall negative misophonic response, but not be germane to the development and maintenance of a misophonia trigger. We propose that the IPR is an integral part of the reinforced learning of misophonia, but there may be other, yet unknown factors that are responsible for the reinforced learning of misophonia. While our clinical cases appear to confirm that the IPR is integral to the development and maintenance of misophonia, more research is required to confirm that the IPR is not simply another symptom of misophonia.

### Possible broader implications for an IPR

We speculate other conditions may sometimes include a reflex similar to the IPR of misophonia. A physical reflex elicited by a phobic stimulus may contribute to the phobic response and maintenance of the phobia. Similarly, a physical reflex might contribute to other conditions such as PTSD, tinnitus, or anxiety. As this is currently unexplored, it may be an important part of other disease processes. Furthermore, the IPR may be a factor supporting the James-Lange theory of emotion ( Cannon, 1987), which proposes that emotions are the result of physiological arousal.

### Research opportunities

This model identifies the need for basic research on stimulus-response (S-R) Pavlovian conditioning and applied misophonia research. S-R Pavlovian conditioning theory was experimentally demonstrated by Donahoe and Vegas (2004) and has received a lot of support. Also, crucially, we find no rebuttals of S-R Pavlovian conditioning theory, and no research studies refuting the findings. One supporting study focused on the role of responses in Pavlovian acquisition and reported that the datasets they analyzed support the response-dependent model, in which CR acquisition is based on the occurrence of the CR rather than the CS-US experiences ( Ghirlanda and Enquist, 2019). Still, fundamental research on S-R conditioning is needed. Research opportunities include investigating the commonly reported learned reflex response to pagers. With the advent of the pager smartphone app, a surgeon can change the alert tone regularly to prevent the development of a conditioned response. This may allow real-life data to be collected on the acquisition of a CR to a repeating, innocuous stimulus. Additional study opportunities include cancer patients’ response to their chemotherapy pump, and study participants’ response to a unique sound that indicates an aversive consequence. This may help us understand how everyday life activities allow misophonia to develop.

Application of this model to applied misophonia research may be even more important. Several studies have reported success in reducing misophonia severity using CBT techniques ( Frank and McKay, 2019; Jager *et al.,* 2021; Lewin *et al.,* 2021; Schröder et al., 2017). Specific components of reducing the IPR and covert mental review may be added to existing protocols to determine additional treatment benefit. Several case studies indicate that reducing the IPR, even when there are comorbid conditions, can reduce misophonia severity ( Dozier, 2015a, 2015c, 2022).

We recommend misophonia treatment studies assess participants’ IPR, because muscle response IPRs (e.g., shoulder flinch) contribute to effective treatment after appropriate muscle relaxation training ( Dozier, 2022), whereas others (e.g., sexual response or intestinal constriction) have made treatment difficult or impossible because these responses cannot be willfully controlled and they are very aversive. This may be an important variable to help understand differences in individual response to treatment. The method of determining the IPR is described by Dozier and Morrison (2017) and can be aided by the free Misophonia Reflex Finder app ( https://apps.apple.com/us/app/misophonia-reflex-finder/id983574804).

We recommend independent research to validate the existence of the IPR of misophonia, which to date has only been identified by Dozier and his collogues. Dozier and Morrison (2017) report varied IPR sensations in a sample of 26 individuals. This supports the existence of somatic responses to misophonic triggers, but it does not show the temporal relationship in the misophonic response chain, and it relied on self-report of participants. Therefore, this study needs to be replicated and expanded. There are likely other ways to validate the existence of the IPR as a typical component of the misophonic response.

Research on the timing of the trigger stimulus, IPR, and emotional response could increase our understanding of the underlying reflex responses that cause the misophonic experience. A pilot study reports an interstimulus response (trigger to IPR) of 200ms for auditory triggers and 350ms for visual triggers ( Dozier *et al.*, 2020). Studies investigating the misophonia emotional response using fMRI have a sample time of 3 seconds, so we currently have no data on the relationship between the muscle response of the IPR and the misophonia emotional response.

We recommend experimental evaluation of treatment protocols based on this model. The uniqueness and simplicity of the Mitchell-Dozier model can provide many opportunities for a wealth of research into the nature and features of misophonia.

Finally, we acknowledge the difficulty of conducting research on the IPR. Our experience and Dozier and Morrison (2017) indicate the physical response of the IPR is different in each person. Therefore, a group study with electromyography contacts on specific muscles or a brain imaging study would likely not show the individualized physical response of each person. Additionally, many responses may be difficult to empirically detect due to the muscle being deep in the body, or for bodily sensations such as feeling numb or a flash of warmth. Furthermore, studies on the IPR and our clinical tests relied predominately on self-report, which has inherent reliability concerns. Even though research on the IPR may be challenging, the significance of such a feature of misophonia makes it worthy of investigation.

## Conclusion

We assert that the Mitchell-Dozier model provides a framework to understanding misophonia based on widely accepted neurological human learning processes.

While the information presented herein is only preliminary supporting evidence for the Mitchell-Dozier model, we believe this model is fundamentally accurate, and provides a coherent, comprehensive theory of misophonia as a conditioned response disorder. Research to validate (or invalidate) the model may be critical to establishing a consensus view of misophonia, which would then facilitate incorporating misophonia into established nosological systems (DSM and/or ICD). Once validated, this model may be considered one possible definition of misophonia. Based on the Mitchell-Dozier model, misophonia is a conditioned aversive response disorder, and we propose that Conditioned Aversive Response Disorder (CARD) is a more appropriate name for this condition than misophonia.

## Data Availability

No data are associated with this article.
