# Supplementary material for: Novel five-phase model for understanding the nature of misophonia, a conditioned aversive reflex disorder
Source: F1000Res. 2023 Oct 4;12:808. Originally published 2023 Jul 11. [Version 3] doi: 10.12688/f1000research.133506.3 (PMC10594049; doi:10.12688/f1000research.133506.3)
Supplement: Peer review history

# Reviewer report: Reviewer response for version 3

**DOI:** 10.5256/f1000research.156528.r212089

**Published:** 2023-11-10

**Authors:** Jordan Norris

**Affiliations:** 1 The University of Oklahoma, Norman, Oklahoma, USA

Thank you to the authors for addressing my comments. I am happy with the current version.

Is the rationale for developing the new method (or application) clearly explained?

Yes

Is the description of the method technically sound?

Yes

Are the conclusions about the method and its performance adequately supported by the findings presented in the article?

Yes

If any results are presented, are all the source data underlying the results available to ensure full reproducibility?

No source data required

Are sufficient details provided to allow replication of the method development and its use by others?

Yes

Reviewer Expertise:

Translational Neuroscience with a focus on neurodevelopment and neurodevelopmental disorders. I have specific expertise in the neurophysiology and clinial phenotypes of misophonia.

I confirm that I have read this submission and believe that I have an appropriate level of expertise to confirm that it is of an acceptable scientific standard.

# Reviewer report: Reviewer response for version 3

**DOI:** 10.5256/f1000research.156528.r213570

**Published:** 2023-10-23

**Authors:** Jeyasakthy Saniasiaya

**Affiliations:** 1 Universiti Malaya, Kuala Lumpur, Malaysia

The authors aim to develop a five-phase model of misophonia from a cognitive-behavioural framework.

 Some suggestions:

1. Do add in epidemiology and risk factors of misophonia.
2. The justification of the proposed five-phase model needs to be more elaborate.
3. Add briefly on misophonia management outcome across the population.
4. The authors conclude that: "We assert that the Mitchell-Dozier model provides a framework for understanding misophonia based on widely accepted neurological human learning processes". This needs to be justified.

Is the rationale for developing the new method (or application) clearly explained?

Yes

Is the description of the method technically sound?

Partly

Are the conclusions about the method and its performance adequately supported by the findings presented in the article?

Partly

If any results are presented, are all the source data underlying the results available to ensure full reproducibility?

Yes

Are sufficient details provided to allow replication of the method development and its use by others?

Yes

Reviewer Expertise:

Otorhinolaryngology, Head and Neck Surgery

I confirm that I have read this submission and believe that I have an appropriate level of expertise to confirm that it is of an acceptable scientific standard.

# Response

**Published:** 2023-10-24

**Authors:** Thomas Dozier

**Affiliations:** Misophonia Institute, Livermore, CA, USA

We are very pleased with the outcome of the latest review and we thank the reviewers for their feedback. We have decided not to make any further changes to the paper. Please see below for details.

 Here are specific responses to the latest review:

 1. This paper is intended to discuss the mechanisms behind misophonia. Page three discusses age of onset and prevalence rates in different populations. We think this is sufficient background information and that adding further details about epidemiology and risk factors (which could be a very long discussion in itself) would distract from our point.

 2. We acknowledge in the Discussion that “additional independent research is needed to validate this model and the effects of each phase as discussed above.” Nevertheless, the model is based on our extensive clinical observations and supported by other Pavlovian conditioning research.

 3. Similar to the above point, this paper is not intended to be a general examination of misophonia. We do discuss misophonia management with reference to our model in the “clinical implications” section, but we do not think it would appropriate to get distracted by a wider discussion of misophonia management.

 4. Here we are rephrasing our comment of the Dozier model, that “it is based on known neurological processes, namely Pavlovian conditioning and the development of learned emotional reflexive responses,” rather than a neurological abnormality or hypothetical process which was supported in the introduction.

# Reviewer report: Reviewer response for version 2

**DOI:** 10.5256/f1000research.154032.r195347

**Published:** 2023-09-14

**Authors:** Jordan Norris

**Affiliations:** 1 The University of Oklahoma, Norman, Oklahoma, USA

First, I want to commend the authors on their important contribution to the young field of misophonia research. The Mitchell-Dozier model is an important step forward in working to standardize conceptualization of misophonia and may prove foundational in exploring misophonia etiology.

 The manuscript details an updated, 5-phase model of misophonia developed through a cognitive-behavior framework building on conditioning theory. The authors describe misophonia as a conditioned response in which trigger stimuli elicit a conditioned physiological response followed by extreme emotions/emotional reactivity. Phases 4 and 5 are comprised of the stress response that follows the emotional response (i.e., limbic activation) and subsequent behaviors individuals with misophonia use to cope. I commend the authors on both their observation of the physiological response/physical reaction associated with trigger exposure and their inclusion of this in their model.

1. Throughout the introduction, where citations are provided, the authors very minimally paraphrase language from cited papers, without integration into their developed theory. While the sentences are not word-for-word taken from the cited references, in some cases they are extremely similar (mainly starting in paragraph 5 – starting with, “There is a lack of consensus on both the etiology of misophonia…”). This lack of integration makes it difficult to determine which ideas presented are the authors' own based on work from the cited papers and which are just presenting directly ideas from other researchers. I would suggest some rewording in the introduction to provide a clearer picture of how these papers and ideas from other researchers support the author’s model.
2. Second, I highly recommend the authors comb through the manuscript for areas where claims are made about misophonia without appropriate citations and add in citations. Given the wealth of information the authors are pulling on to justify their update model, having sources for where their information came from (even if that is from clinical observation) is important. I have provided examples of places in the manuscript where citations are required below but highly suggest the authors make adjustments wherever possible, especially when they say things like “…have been widely documented” and then fail to cite anything.

Some of the following recommendations are nit picky but please hang with me.

- The conclusion of the abstract needs to support the overall goal of the manuscript. I suggest adding more about the model and how it works to support the author’s conclusion about the nature of misophonia. Given this is not an empirical study nor a review article, I highly recommend softening the language with respect to what misophonia is/is not and focusing more on how your model supports the framework of misophonia, as it is described in the literature (i.e., how it builds on the author’s prior findings).
- In paragraph 4 (begins with “ Misophonia is a clinically significant mental health issue…”, could the authors provide more information on the mentioned “community samples” and also for the mentioned “clinical populations” (i.e., which clinical populations).
  - Also, there are prevalence rates in a nonclinical sample reported in Wu *et al.,* 2014 that are worth mentioning here.
- In paragraph 6 (begins with “Jastrebroff and Jastreboff (2002) presented the theory…) the authors mention activation of the autonomic nervous system (ANS) being a central part of Jastrebroff’s theory and that the ANS is not identified as a primary brain area responsible for misophonia. This line may be misleading, as the ANS is technically a peripheral nervous system component, including both the sympathetic and parasympathetic nervous systems, and not a set of brain regions. I would assume the authors meant to refer to the hypothalamus here but don’t know if that assumption is correct - could the authors specify what they mean here.
- In paragraph 8, the authors refer to the emotional response (i.e., anger, irritation, or stress) as an unconditioned stimulus but I believe per conditioning theory that the emotional response is the unconditioned response, not an unconditioned stimulus. I’m aware Palumbo *et al.* (2018) stated this in their manuscript, but I don’t believe it is correct. Namely, if subsequent behavior/coping mechanisms are the conditioned response then the emotional reactivity would be the unconditioned response, regardless. Further, the trigger as the CS and the emotional response as the UR lines up with the next sentence about familial behavior as a US leading to their behavior (the trigger) becoming the CS.
- In paragraph 14 (begins “The current study presents the Mitchell-Dozier model”, somewhat justifies the current model’s deviation from past theoretical models of misophonia but does not provide examples of the broadly recognized characteristics of misophonia that are not reported in the literature on which they are basing this deviation. If these characteristics are not reported earlier in the manuscript, I highly suggest adding them. Common clinical observations are very valuable, and it would add strength to the justification of updating the model. If they are mentioned, I suggest providing an example for continuity and ease of reading.
  - Additionally, this section lacks references and should have at least one to justify the claim that the Mitchell-Dozier model follows accepted cognitive-behavioral explanations of pathology development.
- Paragraph 20 (begins “anticipatory anxiety can present differently…”, needs references. Further, an explanation of why there may be differences (e.g., developmental rationale) would be useful – particularly for those thinking about how to explore the development of misophonia from childhood to adulthood.
- Paragraph 38 (first under phase three): The 3 sentences after the first sentence which introduces the third phase of the model lack proper citations. Please add citations for 1., emotional responses being the primary complaint, and 2.) the variety of emotional responses.

Overall, this was a well-conceived theory. I agree with the authors that this model will support future research and I share their hope that it will produce novel ideas for treatment.

Is the rationale for developing the new method (or application) clearly explained?

Yes

Is the description of the method technically sound?

Yes

Are the conclusions about the method and its performance adequately supported by the findings presented in the article?

Yes

If any results are presented, are all the source data underlying the results available to ensure full reproducibility?

No source data required

Are sufficient details provided to allow replication of the method development and its use by others?

Yes

Reviewer Expertise:

Translational Neuroscience with a focus on neurodevelopment and neurodevelopmental disorders. I have specific expertise in the neurophysiology and clinial phenotypes of misophonia.

I confirm that I have read this submission and believe that I have an appropriate level of expertise to confirm that it is of an acceptable scientific standard, however I have significant reservations, as outlined above.

# Response

**Published:** 2023-09-23

**Authors:** Thomas Dozier

**Affiliations:** Misophonia Institute, Livermore, CA, USA

We appreciate the comments of the reviewer and have attempted to address all recommendations. We have specified that the purpose of the brief summaries of theories of misophonia was to provide context and show that many of the theoretical statements on misophonia were vague. We agree with your concern about the US/UR of Palumbo et al. (2018), but we feel we should state their theory (even if it is incorrect). After the summaries, we compared the three developed theories and indicated similarities and differences to the new model. As recommended, we no longer refer to the ANS as “brain.”  As recommended, we added appropriate citations (19 new references and 40 citations). We revised the abstract to highlight the unique features of this model, which is the primary focus of this manuscript, and we provided more information and references on the prevalence of misophonia. . Finally, we added references and examples to paragraph 14. 

 Again, we appreciate your recommendations and the opportunity to improved the quality of this manuscript.

# Reviewer report: Reviewer response for version 2

**DOI:** 10.5256/f1000research.154032.r194050

**Published:** 2023-08-14

**Authors:** Jadon Webb

**Affiliations:** 1 Bloom Mental Health LLC, Littleton, CO, USA

I was able to review the edits, and these seem thoughtful and appropriate. The authors addressed the use of cautionary/tentative language, and wrote a section on alternative explanations. They also mention the curious possibility of the IPE being present in other conditions (this is unknown). 

 I again thank the authors for their hard work and thoughtful exploration of this new field. They are to be commended for this, and I am happy to support this moving towards indexing.

Is the rationale for developing the new method (or application) clearly explained?

Partly

Is the description of the method technically sound?

Partly

Are the conclusions about the method and its performance adequately supported by the findings presented in the article?

Partly

If any results are presented, are all the source data underlying the results available to ensure full reproducibility?

No source data required

Are sufficient details provided to allow replication of the method development and its use by others?

Partly

Reviewer Expertise:

Misophonia, psychiatry, mental health, anxiety, PTSD

I confirm that I have read this submission and believe that I have an appropriate level of expertise to confirm that it is of an acceptable scientific standard.

# Reviewer report: Reviewer response for version 1

**DOI:** 10.5256/f1000research.146501.r186207

**Published:** 2023-07-27

**Authors:** Jadon Webb

**Affiliations:** 1 Bloom Mental Health LLC, Littleton, CO, USA

This Method article addresses an important, unmet need in the emerging field of misophonia research, and the author should be commended for the hard work of advancing this nascent field.

 The author specifically outlines a five-phase process to explain the development and maintenance of misophonia. This model is based on well-known neuroscience principles including conditioning towards an unpleasant stimulus, and the role of maladaptive psychosocial processes that may reinforce misophonia symptoms, in addition to increasing overall dysfunction.

 A number of researchers have speculated about the disease process that leads to misophonia, and it is clear that as of this writing, there is still no one consensus view for what causes this disorder. Perhaps this should not be surprising, since the understanding of other complex neuropsychological processes, such as depression or anxiety, are also still debated in the details.

 The author specifically proposes in this five phase model the contribution of two elements that are unique and have not been tested or proposed by any other researchers, specifically, the initial physical reflex (IPR) and covert mental review. Of these, the IPR is perhaps the most unique. 

 The author states in the paper: “The IPR has not been acknowledged by misophonia researchers, and it is generally not acknowledged by individuals with misophonia. However, within our clinical setting we find that virtually all of our patients acknowledge the IPR after simple tests are conducted in a treatment session. The other unique phase of this model, covert mental review, is often acknowledged, but the importance of this feature of misophonia in reducing misophonia severity is not appreciated. We find that reducing covert mental review of triggers can reduce the dysfunction of misophonia. Therefore, the unique and critical features of this model have found much support within the clinical setting (Dozier, 2015a, 2015c, 2022); however, more robust research is required to confirm these findings on a larger scale.”

 From the above statement, the author acknowledges that the IPR is not recognized by other researchers and is typically not recognized by patients, at least outside of their own clinical setting. Similarly, covert mental review is not recognized by other groups as an obvious part of misophonia pathology.

 The purpose of research and articles such as this, is to advance new hypotheses that may revolutionize our approach to diagnosis and treatment. However, until these proposed mechanisms are validated by empiric evidence, and replicated by outside researchers, caution must be exercised in how much confidence we can have in them. 

 The language style in this paper is enthusiastic, but also appears at times to sound almost insistent that this model is solid, while citing only evidence from within the author’s own lab. To the outside reader, this raises the obvious question of why elements such as the IPR have not gained more widespread recognition if there is obvious explanatory, diagnostic, or treatment utility to it.

 It is certainly possible that the rest of the field simply has not caught up yet to this new truth. We know that this sometimes happens. However, it is incumbent on those developing the new model to take an appropriately tentative stance, and when possible, follow a classically scientific approach, which is to be the worst critic, and seek to falsify your own proposed hypothesis and show this work - not to try to convince the outside reader that the model is good.

 This paper is well researched, the author is an expert that clearly understands misophonia as well as any other expert in the world, and this proposed model has a lot of potential. However, I suggest that the author attempt to change the language to be more classically scientific, taking a more critical look at this hypothesis, using more tentative language, and clearly highlighting the areas of uncertainty and weakness. And, proposing alternative explanations to this model that can be tested. 

 For example, as previously mentioned, perhaps the IPR is not always (or ever?) a causal agent in the chain of misophonia pathology. Perhaps it is an incidental observation, or a tangential symptom that can be observed, may sometimes be annoying or add in to the overall pathology, but does not necessarily *cause* the subsequent emotional response and reinforced learning.

 It is one thing to note that misophonia patients often seem to have a muscle/somatic reflex to a trigger (which is a fantastic observation in its own right). It is another to propose that it is a necessary, causal step in the disease process. This is a huge claim, and there are no doubt many alternative hypotheses for if/how this reflex plays a role. Again, this need to be very clearly outlined so that the hypothesis can be rejected, and so that other labs could clearly understand how to test it. 

 I suggest it should almost be written in a format that a graduate student trying to find a thesis project could use as their guiding document. 

 Of note: it may also be of interest to speculate if the IPR is a unique signature for only misophonia, or if it may also be an undiscovered part of other disease processes, such as phobias or PTSD.

Is the rationale for developing the new method (or application) clearly explained?

Partly

Is the description of the method technically sound?

Partly

Are the conclusions about the method and its performance adequately supported by the findings presented in the article?

Partly

If any results are presented, are all the source data underlying the results available to ensure full reproducibility?

No source data required

Are sufficient details provided to allow replication of the method development and its use by others?

Partly

Reviewer Expertise:

Misophonia, psychiatry, mental health, anxiety, PTSD

I confirm that I have read this submission and believe that I have an appropriate level of expertise to confirm that it is of an acceptable scientific standard, however I have significant reservations, as outlined above.

# Response

**Published:** 2023-08-01

**Authors:** Thomas Dozier

**Affiliations:** Misophonia Institute, Livermore, CA, USA

We appreciate the comments of the reviewer and have attempted to address all recommendations. We acknowledge the enthusiastic language and have toned it down. Twelve statements that originally read as a 'fact' were change to read as a proposal or characteristic of the model. We added sections on alternate explanations and on implications of the IPR for other disorders. We acknowledge the comment to expand recommendations for research that would allow the model to be tested/rejected, but we have not identified such research methods. So, we added a paragraph on the issues that make research on the IPR difficult. In fact, for the past year we have looked for researchers that Misophonia Institute could fund for research on this model, but have yet to identify an institution interested in studying this model, even if funding is provided.
